# Neural masses and fields: modeling the dynamics of brain activity

**DOI:** 10.3389/fncom.2014.00149

**Published:** 2014-11-17

**Authors:** Dimitris Pinotsis, Peter Robinson, Peter beim Graben, Karl Friston

**Affiliations:** ^1^The Wellcome Trust Centre for Neuroimaging, University College LondonLondon, UK; ^2^School of Physics, University of SydneyNSW, Australia; ^3^Center for Integrative Brain Function, University of SydneyNSW, Australia; ^4^Brain Dynamics Centre, Westmead Millennium InstituteWestmead, NSW, Australia; ^5^Institut für deutsche Sprache und Linguistik, Humboldt-Universität zu BerlinBerlin, Germany

**Keywords:** integro-differential equations, neural field theory, neural masses, anaesthesia, electrophysiology, electroencephalogram, oscillations, neural disorders

Biophysical modeling of brain activity has a long and illustrious history (Ermentrout, 1998; Deco et al., 2008; Coombes, 2010) and has recently profited from technological advances that furnish neuroimaging data at an unprecedented spatiotemporal resolution (Guillory and Bujarski, 2014; Sporns, 2014). Neuronal modeling is a very active area of research, with applications ranging from the characterization of neurobiological and cognitive processes, (Jirsa, 2004b,a; Bojak and Liley, 2005; Phillips and Robinson, 2009; Rolls and Treves, 2011) to constructing artificial brains *in silico* and building brain-machine interface and neuroprosthetic devices, e.g., Einevoll et al., 2013; Whalen et al., 2013. Biophysical modeling has always benefited from interdisciplinary interactions between different and seemingly distant fields; ranging from mathematics and engineering to linguistics and psychology. This Research Topic aims to promote such interactions by promoting papers that contribute to a deeper understanding of neural activity as measured by fMRI or electrophysiology.

In general, mean field models of neural activity can be divided into two classes: neural mass and neural field models. The main difference between these classes is that field models prescribe how a quantity characterizing neural activity (such as average depolarization of a neural population) evolves over *both space and time* as opposed to mass models, which characterize activity over time only; by assuming that all neurons in a population are located at (approximately) the same point. This Research Topic focusses on both classes of models and considers several aspects and their relative merits that: span from synapses to the whole brain; comparisons of their predictions with EEG and MEG spectra of spontaneous brain activity; evoked responses, seizures, and fitting data—to infer brain states and map physiological parameters.

## Extensions of mean field models and modeling of anaesthetic action

Some of the contributions consider extensions of neural mass and field models and their relation with other classes of models, with a particular focus on modeling the action of anesthetics:

Liley and Walsh (2013) hypothesize that fast-slow dynamics, as exhibited in individual neuron bursting, dynamically underpins electroencephalographic bursting. They are able to modify a well-known mean field model of the electroencephalogram by adding slow variables. This can be seen as a metaphor for anesthetic action, and allows them to produce a wide variety of burst-like activities. Bojak et al. (2013) look at quantitative modulations of EEG activity resulting from manipulating the anesthetics ketamine and propofol. They are able to determine parameter ranges that produce observed modulations in alpha peak frequency, and predict antagonistic drug interactions. The action of anesthetics, in the context of mean field models, is also discussed by Hutt (2013). The author considers a linear neural population model and presents an analytic derivation of the power spectrum that depends on propofol concentration. He then explains the anesthetic-induced power increase in neural activity as a result of an oscillatory instability and derives conditions under which the power peak shifts to higher frequencies, as observed experimentally in EEG.

The roles of neural mass, conductance based, and neural field models in dynamic causal modeling (DCM) are reviewed and explored by Moran et al. (2013). These authors show that such models can reproduce the characteristics of spectra and evoked responses observed empirically, with conductance based models having a richer repertoire of dynamics than neural mass models. Neural field models are able to capture lateral interactions and allow detailed analysis of structure-function relationships in the cortex.

Modolo et al. (2013) discuss neural masses designed to study the interaction between power-line magnetic fields and brain activity. They demonstrate that EEG alpha power could be modulated by weak membrane depolarization induced by the exposure to power-line magnetic fields and explore the role of input noise on EEG power modulation. A different use of neural fields is presented in Wright and Bourke (2013). These authors propose that both synchronous firing of neurons—and their competition for limited metabolic resources during neural development—lead to ultra-small-world neural networks. These networks then exhibit Möbius strip-like topologies that putatively reflect structure in striatal visual cortex.

The contribution of Pinotsis et al. (2013) introduces a conductance-based neural field model combining biologically realistic synaptic dynamics with neural field equations. These authors demonstrate that both the evoked responses and induced responses show qualitative differences depending on the chosen model, either neural mass or neural field.

## Explaining activity observed in neurological disorders and cognitive tasks

Other articles in this Research Topic relate to the use of field models to explain aberrant neural activity and dynamics recorded during cognitive tasks: Kerr et al. (2013) integrate field and network models in a multiscale model. This allows the authors to reveal alterations in cortical information flow between normal subjects and Parkinsonian patients, quantified by a decrease in Spectral Granger Causality between cortical layers in the beta frequency.

Frequency-dependent effects in deep brain stimulation in epileptic patients are studied using computational modeling and intracerebral EEG data in Mina et al. (2013). This paper describes the biophysics of direct stimulation of the thalamic compartment of an established thalamocortical model at the cellular level. It also demonstrates that low-frequency and high-frequency stimulation are beneficial for suppressing epileptic seizures, but that intermediate frequencies favor thalamic oscillations and entrain epileptic dynamics, rather than suppressing them.

Bhattacharya's paper (Bhattacharya, 2013) also focuses on explaining brain oscillations in sickness and health. The author replaces the “alpha function” approximation for synaptic transmission by a kinetic framework of neurotransmitter and receptor dynamics. The results are compared with experimental studies and shown to be consistent; they also lead to an order of magnitude improvement in simulation times compared to the alpha function approach commonly adopted in neural mass models.

In Srinivasan et al. (2013) the authors study an important phenomenon observed in EEG data, called phase-amplitude coupling, and show how it can be modeled using classical Wilson and Cowan equations. This is not only a mathematical exercise; it allows for a description of important top-down influences on local networks as a result of behavioral (e.g., attentional) or pharmacological manipulations—and fits well with results from the animal and human literature.

In another paper, Robinson et al. (2012) explore the functional neuroimaging measurements required to characterize neocortical activity. In particular, they show that some state changes can occur independently of changes in average amplitude, power, or metabolic indexes. They then introduce a new measure of complexity that can uncover the corresponding dynamical structure inherent in cortical activity, which would otherwise be difficult or impossible to detect.

Finally, beim Graben and Rodrigues (2012) reduce a simplified 3-compartment neuron model into a leaky integrate-and-fire (LIF) model describing spiking dynamics and derive an observation model for dendritic dipole currents in extracellular space that contributes to the local field potential (LFP) of a neural population. They introduce a new way to predict LFPs in network simulations involving only single-compartment neurons and compare their method with the results of an earlier approach (Mazzoni et al., 2008).

## Theory of mean field models

In addition to papers focussing on applications, this Research Topic includes theoretical papers studying the mathematical aspects of mean field theory: Bressloff and Wilkerson (2012) study rigorous aspects of field models using an off-centered connectivity kernel that can serve as a model for direction selectivity. They prove the existence and stability of stimulus-induced activity pulses assuming a Heaviside firing rate function and including spatiotemporal noise. These authors conclude that freely moving pulses are more sensitive to multiplicative noise than stimulus-locked pulses.

In Gray and Robinson (2013), the authors address an important issue in the literature on neural networks; that is, what are the effects of time delays and dendritic time constants on the stability constraints of the network dynamics. They approach this question from the perspective of their prior work, in particular the Robinson, Rennie Wright model (RRW). Within this framework, they introduce a constant time delay and then systematically analyze the stability of a network state as a function of time delays and other parameters.

Roy and Jirsa (2013) show how a novel neurocomputational unit model qualitatively captures the complex dynamics exhibited by a full network of parabolic bursting neurons. The reduced representation is mathematically tractable and allows the authors to derive appropriate boundary conditions for various dynamical regimes. This approach sheds light on the role of slow oscillations for determining the global behavior of brain networks. Finally, Augustin et al. (2013) examine how the dynamics of adaptation currents contribute to spike rate oscillations in recurrent neural networks. They find frequency-dependent effects that can have roles in generation of specific frequencies and selective signal propagation.

The above anthology of papers provides illustrative examples of recent advances in biophysical modeling. This line of work speaks to the hope that such models may help explain neural dynamics that underpin disorders like epilepsy or Parkinson's disease as well as normal functions like attention or working memory; an endeavor we hope the articles in this volume will progress.

### Conflict of interest statement

The authors declare that the research was conducted in the absence of any commercial or financial relationships that could be construed as a potential conflict of interest.
